# Asymptomatic Congenital Lung Malformations: Timing of Resection Does Not Affect Adverse Surgical Outcomes

**DOI:** 10.3389/fped.2020.00035

**Published:** 2020-02-14

**Authors:** Vincent Duron, Ariela Zenilman, Cornelia Griggs, Jennifer DeFazio, Jessica C. Price, Weijia Fan, Matthew Vivero, Juliana Castrillon, Maggie Schmaedick, Emaad Iqbal, Steven Rothenberg

**Affiliations:** ^1^Department of Pediatric Surgery, NewYork-Presbyterian Morgan Stanley Children's Hospital, New York, NY, United States; ^2^Columbia University Mailman School of Public Health, New York, NY, United States; ^3^Vagelos College of Physicians and Surgeons, Columbia University Irving Medical Center, New York, NY, United States; ^4^Department of Pediatric Surgery, Rocky Mountain Hospital for Children at Presbyterian/St. Luke's Medical Center, Denver, CO, United States

**Keywords:** thoracoscopic surgery, congenital lung and airway malformation, surgical timing, lung resection surgery, congenital pulmonary adenomatous malformations, bronchopulmonary sequestration, pediatric surgery

## Abstract

**Background:** Optimal timing for resection of asymptomatic congenital lung malformations (CLMs) remains controversial. The aim of this study is to define optimal timing for surgical intervention of patients with CLMs and define clinical variables that affect surgical outcomes.

**Methods:** An IRB-approved retrospective analysis was conducted for patients undergoing surgery for CLMs between 2012 and 2017. Subjects were divided into cohorts based on timing of operative intervention. “Early intervention” was defined as surgery within 4 months of birth; “intermediate intervention”—between 4 and 6 months; and “late intervention”−6–12 months. Surgical outcomes including intraoperative estimated blood loss (EBL), surgical time, post-operative pneumothorax, length of time chest tube stayed in, and hospital length of stay were compared among the three groups using Fisher's exact test or Chi-squared test for categorical variables and one-way analysis of variance test for continuous variables.

**Results:** We analyzed 63 patients who underwent surgery for CLM. There were no significant differences in baseline characteristics. Timing of surgery did not significantly correlate with post-operative outcomes. Specifically, there was no difference in operative time, EBL, post-operative pneumothorax, or length of hospital stay among the early, intermediate, and late intervention groups. Even after controlling for cyst-volume ratio (CVR), timing of surgery still did not affect post-operative outcomes.

**Conclusions:** Surgical outcomes for resection of CLMs are not significantly affected by timing of surgery. We advocate for early intervention to decrease the incidence of associated complications that can occur with later intervention.

## Introduction

Congenital pulmonary airway malformation (CPAM) are hamartomatous masses of the airway and lungs with cystic and adenomatous features believed to result from abnormal branching development of the lung in utero. Bronchopulmonary sequestrations (BPS) are a type of congenital lung lesion in which the lung tissue has no connection to the tracheobronchial tree and has abnormal systemic arterial supply. Hybrid lesions contain elements of CPAM and BPS (1, 2). Some surgeons elect to observe these lesions and intervene on them if symptoms develop. Many surgeons operate on these lesions during infancy to prevent infection, malignant transformation and to allow compensatory lung growth (3–6). Optimal timing for resection of asymptomatic congenital lung malformations (CLMs)—congenital pulmonary airway malformation and bronchopulmonary sequestration—remains controversial (7). Early intervention decreases the risk of respiratory infections and the risk of malignant transformation. It also may allow more time for compensatory lung growth, which is thought to continue during infancy (8, 9). Air-leak, pleural effusion, development of respiratory illness, and chylothorax have been documented in patients who undergo later intervention (10). Others prefer later intervention when the infant is bigger and the surgery may be safer. The purpose of this study is to (1) define optimal timing for surgical intervention of patients with CLMs and (2) define clinical variables that affect surgical outcomes.

## Materials and Methods

An IRB-approved retrospective analysis was conducted for patients undergoing surgery for asymptomatic CLMs between 2012 and 2017. Over a 5 year period, 63 patients under 1 year of age underwent surgical intervention for congenital lung malformations at our institution, the NewYork-Presbyterian Morgan Stanley Children's Hospital. Preoperative clinical characteristics included sex, gestational age, birth weight, cyst-volume ratio (CVR), gestational complications. Post-surgical evaluation included estimated intraoperative blood loss (EBL), surgical time, post-operative pneumothorax, and hospital length of stay. “Early intervention (EI)” was defined as surgery within 4 months of birth; “intermediate intervention (IMI)”—between 4 and 6 months; and “late intervention (LI)”−6–12 months. Patients were categorized according to time of intervention. All children underwent thoracoscopic resection. No patient required conversion to open thoracotomy.

Descriptive statistics were reported for baseline characteristics. Categorical variables were reported with frequency and percentage, while continuous variables were reported as median with first quantile and third quantile. Fisher's exact test was used for categorical variables while Kruskal-Wallis test was used for continuous variables when comparing among 3 groups. As CVR has been shown to correlate with disease severity, specifically incidence of hydrops and need for fetal intervention as well as possibly postnatal respiratory symptoms from literature (11–14), models adjusted for CVR were fit using linear regression to assess the association between outcomes of interest and the main predictor.

This study was conducted in accordance with Columbia University Medical Center human research protection guidelines and with protocol approval from IRB-AAAR5966. Protocol approval included approved waiver of consent compliant with federal U.S. Department of Health & Human Services 45CFR46.116(d) criteria.

## Results

From February 2012 to February 2017, 66 patients underwent thoracoscopic resection of a CLM. Patients with sub-diaphragmatic and multi-lobar lesions were excluded to generate a more homogenous and therefore comparable population. SR, our senior surgeon, was involved in all surgeries. The surgeries were performed in an academic center with pediatric surgery fellows. Table 1 summarizes prenatal and pre-operative characteristics (Table 1A–categorical variables, Table 1B—continuous variables). There was no difference in baseline characteristics between the three groups. Median birth weight was 2.9 kg (2.7–3.5 kg), with median gestational age at birth of 38.0 weeks (37.0–39.0 weeks). Lesions were right sided in 52% of patients. A feeding vessel was present in 52% of lesions. Median CVR was 0.49 (0.22–0.83), with 1.6% having a CVR >1.6 (classified as high risk).

**Table 1A:** Categorical prenatal and pre-operative characteristics.

**Variables**		**EI (*n* = 22) N (%)**	**IMI (*n* = 20) N (%)**	**LI (*n* = 21) N (%)**	***p*-value**
Gender					0.951
	Female	11 (50%)	11 (55%)	11 (52%)	
	Male	11 (50%)	9 (45%)	10 (48%)	
Feeding vessel					0.232
	No	8 (36%)	11 (65%)	9 (47%)	
	Yes	14 (64%)	6 (35%)	10 (53%)	
	N-unidentified	0	3	2	
Lesion side					0.299
	Left	10 (45%)	8 (40%)	13 (62%)	
	Right	12 (55%)	12 (60%)	8 (38%)	
Lobe affected					0.247
	LLL	10 (46%)	7 (35%)	10 (48%)	
	LUL	0 (0%)	1 (5%)	3 (14%)	
	RLL	11 (50%)	11 (55%)	6 (29%)	
	RML	0 (0%)	0 (0%)	0 (0%)	
	RUL	1 (5%)	0 (0%)	2 (10%)	

*EI, Early intervention group; IMI, Intermediate intervention group; LI, Late intervention group; N-unidentified, Number of patients with no data recorded for variable; LLL, Left lower lobe; LUL, Left upper lobe; RLL, Right lower lobe; RML, Right middle lobe (primarily); RUL, Right upper lobe*.

**Table 1B:** Continuous prenatal and pre-operative characteristics.

**Variables**	**EI (*n* = 22)** **Median (Q1–Q3)**	**IMI (*n* = 20)** ** Median (Q1–Q3)**	**LI (*n* = 21)** ** Median (Q1–Q3)**	***p*-value**
Birth weight Z-score (GA adjusted)	−0.92 (−1.06, 0.07)	−0.10 (−0.91, 0.78)	0.40 (−0.59, 0.71)	0.082
Birth weight (actual, kg)	2.86 (2.61–2.92)	3.14 (2.89–3.50)	3.12 (1.87–3.58)	0.283
Birth weight: N-unidentified	5	3	7	–
Gestational age, weeks	37.5 (37.0–38.2)	38.3 (38.0–40.0)	38.0 (34.6–39.1)	0.171
GA: N-unidentified	0	2	4	–
CVR	0.49 (0.28–1.08)	0.55 (0.40–1.05)	0.30 (0.20–0.55)	0.423
CVR: N-unidentified	11	13	12	–

*EI, Early intervention group; IMI, Intermediate intervention group; LI, Late intervention group; GA, Gestational age in weeks; CVR, Cyst volume ratio; N-unidentified, Number of patients with no data recorded for variable*.

Operative characteristics and post-operative outcomes were categorized by subgroup of early, intermediate, or late intervention (Table 2). Mean operative time and estimated blood loss were not different between groups (*p* = 0.62, 0.48). Rare complications included transfusion requirement due to bleeding (3.2%), chylous chest tube drainage (1.6%), reoperation (1.6%), and reinsertion of chest tube (1.6%).

**Table 2:** Operative and post-operative characteristics.

**Variables**	**EI (*n* = 22)** ** Median (Q1–Q3)**	**IMI (*n* = 20)** ** Median (Q1–Q3)**	**LI (*n* = 21)** ** Median (Q1–Q3)**	***p*-value**
EBL (cc)	5.00 (1.25–10.00)	3.50 (0.75–10.00)	10.00 (2.5–15.00)	0.480
EBL: N-unidentified	0	0	2	–
Hospital LOS (days)	2.00 (1.00–3.00)	2.00 (1.00–2.00)	2.00 (1.00–2.00)	0.316
LOS: N-unidentified	3	0	1	–
Chest tube duration (days)	1.53 ± 1.02	1.85 ± 0.99	1.61 ± 1.24	0.129
Chest tube duration: N-unidentified	4	1	2	–
Operative time (min)	208.39 ± 69.26	178.95 ± 51.82	185.14 ± 78.64	0.623

*EI, Early intervention group; IMI, Intermediate intervention group; LI, Late intervention group; EBL, Intraoperative estimated blood loss (cc); Hospital LOS, Hospital length of stay (days); Chest tube duration– Length of time chest tube in place (days); N-unidentified, Number of patients with no data recorded for variable*.

Of the post-operative outcomes analyzed (Table 2), results again showed no significant difference between each group. The hospital length of stay, incidence of pneumothorax after post-operative day one, and length of time the chest tube stayed in place were all similar among the three groups. Complications were then controlled for cyst-volume ratio, and again no significance was seen between groups for operative time (*p* = 0.61), estimated blood loss (*p* = 0.09), chest tube duration (*p* = 0.12), or hospital length of stay (*p* = 0.57) (Table 3). Two specimen were found to have potentially malignant lesions on pathologic examination—one had bronchioalveolar carcinoma (BAC) and the other pleuropulmonary blastoma (PPB). Both were considered early stage or premalignant and did not need further treatment after resection.

**Table 3:** Post-operative characteristics controlled by cyst-volume ratio.

**Variables**	**Parameter**	**Estimate**	**Standard Error**	***t*-value**	**Pr > |t|**
EBL (cc) *n* = 26					
	Intercept	8.26	2.77	2.98	0.0069
	Cohort (ref: LI)				0.0908
	Cohort EI	−4.55	3.57	−1.27	0.2157
	Cohort IMI	−9.15	3.95	−2.32	0.0303
	CVR	5.17	3.05	1.70	0.1040
Hospital LOS (days) *n* = 25					
	Intercept	1.58	0.32	4.94	<0.0001
	Cohort (ref: LI)				0.5727
	Cohort EI	0.34	0.41	0.81	0.4243
	Cohort IMI	0.47	0.45	1.03	0.3160
	CVR	0.13	0.36	0.36	0.7229
Chest tube duration (days) *n* = 24					
	Intercept	0.90	0.18	4.97	<0.0001
	Cohort (ref: LI)				0.1193
	Cohort EI	0.11	0.23	0.46	0.6488
	Cohort IMI	0.55	0.27	2.05	0.0538
	CVR	0.30	0.21	1.48	0.1550
Operative time (min) *n* = 27					
	Intercept	170.65	27.06	6.31	<0.0001
	Cohort (ref: LI)				0.6146
	Cohort EI	8.54	34.01	0.25	0.8039
	Cohort IMI	−26.18	37.99	−0.69	0.4976
	CVR	20.06	29.79	0.67	0.5074

*EI, Early intervention group; IMI, Intermediate intervention group; LI, Late intervention group; EBL, Intraoperative estimated blood loss (cc); Hospital LOS, Hospital length of stay (days); Chest tube duration– Length of time chest tube in place (days)*.

## Discussion

We performed a retrospective review of our experience with thoracoscopic resection of asymptomatic congenital lung lesions, including CPAM, BPS, and hybrid lesions. Controversy exists in the field whether to resect CLMs in patients who appear to be asymptomatic at birth. Concerns include malignant transformation of the lesion if resection is declined or delayed, as well as increased rates of respiratory infections (10). A strong argument exists for resection before the development of symptoms, because multiple meta-analyses have shown that patients who undergo surgery after development of respiratory symptoms are more likely to experience post-operative complications. Kapralik et al. (4) examined one non-randomized prospective and eight retrospective studies for a total of 168 patients who were asymptomatic at birth. Of these, 41% elected for prophylactic surgery. Sixty-four (64.3%) patients who opted out of surgery at birth eventually required surgery due to later development of symptoms. Total post-operative complications were significantly higher in patients who delayed surgery until after the development of symptoms as compared to those who underwent prophylactic resection while still asymptomatic (odds ratio 4.59, 95% confidence interval between 1.40 and 15.11, *p* = 0.01). These authors hypothesized that exposure to lung infection could be the cause of increased surgical complications, because of the inflammation and scarring of lung tissue that occurs after infection, even though these patients were not infected at the time of the procedure. It has been noted in many studies that surgery after development of symptoms tends to have poorer outcomes than prophylactic surgery. Another study, Style et al. (15) looked specifically at age at the time of resection. Surgeries that occurred before 4 months of age were defined as “early” and those that occurred after 4 months of age were defined as “late,” rather than using absence or presence of symptoms to compare groups. This study found a slight but insignificant increase in complications in resection that occurred after 4 months of age when compared to earlier resection. Complications included air-leak and pleural effusion, development of respiratory illness and one chylothorax. The authors also compared length of operation and length of hospital stay. Operation time was on average 27 min shorter (*p* = 0.05) in earlier resection, but there was no significant difference in length of hospital stay. Note that this study included many congenital pulmonary lesions, CPAM making up a small proportion of cases studied. However, the authors conclude more generally about asymptomatic lung lesions that early resection does not appear to pose a risk and in fact may offer some benefit over delaying the procedure. The authors propose that the 4 month window is significant because of the retention of maternal passive immunity in children under 4 months, which could be protective against post-surgical infectious complications. This study reinforces findings of a previous study by Jelin et al. (16) in which they demonstrated that early resection is equally safe to later resection of CPAM and earlier resection is associated with a decreased operative time.

These studies seem to suggest that the risks of prophylactic surgery (early exposure to anesthesia, infection risk) are not enough to outweigh the risks of an expectant approach (pneumonia and lung infection risk, possibility of malignant transformation) (17). Many patients will eventually become symptomatic and require surgery, and these later surgeries are more dangerous and limit the possibility of compensatory lung tissue growth. Therefore, the general recommendation is resection. A few studies have attempted to ascertain the likelihood that an asymptomatic child will eventually develop symptoms. A systematic review of 41 reports including 1,070 cases found only 3.2% of patients who were antenatally diagnosed and asymptomatic at birth became symptomatic, at a median age of 6.9 months (18). Surgery in symptomatic patients was found to carry a 2-fold greater risk of complications than patients who underwent elective surgery, consistent with previous research. The authors note that most studies are limited by relatively short follow-up periods, and suggest that a follow-up study of at least 10 years would be needed to fully appreciate the long-term effects of perinatal surgical or conservative treatment. Other studies have confirmed that although only 5% will become symptomatic in the first 5 years of life, symptom manifestation occurs at a median age of 7 months, arguing for early intervention even if asymptomatic (4, 11, 19). Reported pathologic conversion is a serious concern in this population as well. Bronchioalveolar carcinoma (BAC) is a very rare lung tumor, usually diagnosed at a mean age of 69 years, however there have been reports in children who underwent resection of CLMs, arguing for early prophylactic resection to prevent BAC later in life (11). One of our patients was found to have BAC and another was found to have pleuropulmonary blastoma on pathologic examination. Thankfully both were found to be early stage and pre-malignant. They have undergone close surveillance and have not required further treatment beyond resection. Previous studies have also shown that earlier age at lung resection, do show improved lung hyperplasia and better ventilatory function (15, 20).

This study is limited as a single center retrospective study. The retrospective design may introduce additional bias, and single center trials may overestimate treatment effect compared with multicenter trials (21). Furthermore, results may not be generalizable to all medical centers as the operative environment, post-operative intensive care, and patient population may differ based on institution, and all surgeries took place in the same center. Conversely, the findings are streamlined by the fact that one surgeon, SR was involved in all surgeries, which may aid in eliminating variability in operative technique. In addition, our results advocating for earlier resection do concur with other similar studies conducted at other institutions as previously discussed (4, 15, 16, 20).

This study confirms the findings of earlier studies that early resection of asymptomatic lung lesions has similar intra-operative and post-operative outcomes than later resection. Over the study period, we have shifted our practice to operating earlier and now offer surgery routinely at 3 months of age. We believe that thoracoscopic pulmonary surgery is technically easier in younger infants mainly because there has been less inflammation due to either clinical or subclinical respiratory infections causing hilar lymphadenopathy and adding complexity to the surgery. Earlier surgery decreases the total time prior to resection where patients can potentially contract respiratory infections or develop symptoms, which at the time of resection are known to be associated with post-op complications and reduced future lung function (4, 18). Also, as is evidenced by our experience and other case reports, malignant or pre-malignant lesions have been detected in resected lung, including in this study a patient with resection at <4 months. Finally, although the evidence is mixed, the theoretical compensatory lung growth that occurs in infants may benefit from earlier resection (22, 23).

In conclusion, we present a single-institution study of infants undergoing thoracoscopic resection of congenital lung lesion. Our findings support other studies that earlier resection has similar outcomes as later resection and may contribute long-term benefit to these patients. Long-term studies are warranted to confirm these findings.

## Data Availability Statement

The dataset analyzed for this article is not publicly available in order to comply with Columbia University Irving Medical Center Human Research Protection and Institutional Review Board standards, and with national Health Insurance Portability and Accountability Act laws. Requests for access to limited de-identified data should be directed to Vincent Duron, MD at vd2312@cumc.columbia.edu.

## Ethics Statement

The studies involving human participants were reviewed and approved by Columbia University IRB-AAAR5966. Written informed consent from the participants' legal guardian/next of kin was not required to participate in this study in accordance with the national legislation and the institutional requirements.

## Author Contributions

SR and VD conceived and designed the study. SR, VD, JD, CG, MV, and EI performed surgeries. SR, AZ, CG, JD, MV, MS, and EI acquired the data. AZ, WF, EI, and JC analyzed the data. VD, AZ, CG, JP, and JC drafted the tables and manuscript. All authors read and approved the final manuscript.

### Conflict of Interest

The authors declare that the research was conducted in the absence of any commercial or financial relationships that could be construed as a potential conflict of interest.
